# Age and gender effect on alexithymia in large, Japanese community and clinical samples: a cross-validation study of the Toronto Alexithymia Scale (TAS-20)

**DOI:** 10.1186/1751-0759-1-7

**Published:** 2007-03-06

**Authors:** Yoshiya Moriguchi, Motonari Maeda, Tetsuya Igarashi, Toshio Ishikawa, Masayasu Shoji, Chiharu Kubo, Gen Komaki

**Affiliations:** 1 Department of Psychosomatic Research, National Institute of Mental Health, National Center of Neurology and Psychiatry, 4-1-1 Ogawa-higashi Kodaira-City, 187-8551, Tokyo, Japan; 2 College of Art and Design, Joshibi University of Art and Design, 1900 Asamizodai, Sagamihara, Kanagawa, 228-8538, Japan; 3 Department of Psychosomatic Medicine, Kohnodai Hospital, National Center of Neurology and Psychiatry, 1-7-1 Kohnodai, Ichikawa, Chiba 272-8516, Japan; 4 Department of Psychosomatic Medicine, Graduate of Medical Sciences, Kyushu University, 3-1-1 Maidashi, Higashi-ku, Fukuoka 812-8582, Japan

## Abstract

**Background:**

The construct validity of alexithymia and its assessment using the 20-item Toronto Alexithymia Scale (TAS-20) in Japan is unknown. Low reliability has been found for the third factor of the TAS-20 in some cultures, and the factor structure for psychosomatic disorder patients has not been adequately investigated. Although alexithymia most likely has certain developmental aspects, this has infrequently been investigated.

**Methods:**

The newly-developed Japanese TAS-20 was administered to a normative sample (n = 2,718; 14–84 y.o.), along with the NEO Five-Factor Inventory (NEO-FFI) for cross validation. Psychosomatic patients (n = 1,924, 12–87 y.o.) were tested to evaluate the factor structure in a clinical sample. College students (n = 196) were used for a test-retest study. Internal reliability and consistency were assessed, and the factorial structure was evaluated using confirmatory and exploratory factor analyses for both the normative and the clinical samples. The correlations between the TAS-20 and the NEO-FFI factor scores were evaluated. Age-related and gender differences in the TAS-20 were explored using analysis of variance in the normative sample.

**Results:**

The original three-factor model of the TAS-20 was confirmed to be valid for these Japanese samples, although a 4-factor solution that included negatively keyed items (NKI) as an additional factor was more effective. Significant correlations of the TAS-20 with the NEO-FFI were found, as has been previously reported. Factor analyses of the normative and patient samples showed similar patterns. The TAS-20 total, difficulty in identifying feelings (DIF), and difficulty in describing feelings (DDF) scores were high for teenagers, decreased with age, and from 30s did not change significantly. In contrast, externally oriented thinking (EOT) scores showed an almost linear positive correlation with age. DIF scores were higher for females, while EOT scores were higher for males, without any interaction between gender and age differences.

**Conclusion:**

The original three-factor concept of the TAS-20 was generally supported for practical use. Age-related differences in TAS-20 scores indicate developmental aspects of alexithymia. Alexithymia is made up of two components with different developmental paths: DIF/DDF and EOT.

## Background

Alexithymia is a disturbance in affective and cognitive functioning [1] and a deficit in emotional regulation [2]. The twenty-item Toronto Alexithymia Scale (TAS-20) is a widely used and validated self-report questionnaire for measuring the severity of alexithymia [3,4] that was constructed with a three-factor structure: (a) difficulty identifying feelings (DIF); (b) difficulty describing feelings to others (DDF); and (c) externally-oriented thinking (EOT). Although the factor structure of the TAS-20 was originally developed in English [3,4], the TAS-20 has been translated into many languages and validated in many cultures [4-9]. Replication of this three-factor model, however, has not been done for a large Japanese sample. Furthermore, the third factor of the TAS-20 has been reported to be lacking in reliability in some cultures where English is not the primary language [6,10-12], hence the need for further examination of the factor structure of alexithymia in non-English speaking countries.

The relationship between the TAS-20 and the NEO Five-Factor Inventory (NEO-FFI), a well-validated personality inventory, has been consistently documented in the English speaking world, and thus can provide a comparison for the validity of the TAS-20 in another culture. The NEO-FFI has been cross-validated for the Japanese culture [13,14]. Thus a comparison of Japanese results on the TAS-20 with the NEO-FFI could indicate whether or not alexithymia is a personality construct that transcends cultural boundaries.

A Japanese edition of the TAS-20 was recently developed by Komaki et al. [15]. The high correlation between the Japanese TAS-20 and the Structured Interview of the Beth Israel Hospital Psychosomatic Questionnaire (SIBIQ) for alexithymia has reinforced the convergent validity of the Japanese TAS-20 [16]. However, a larger sample with wider age-range would be preferable for validation of the Japanese TAS-20 with a normative population data set and concurrent (criterion-related) validity using another concomitant measurement targeting related factors other than alexithymia (e.g., the NEO-FFI).

Only non-clinical samples were used in most of the studies investigating the factor structure of the TAS-20. However, alexithymia has a clinical aspect because it is often found to be higher among patients, therefore it is of interest and important to ensure that the suggested factor structure is also valid in clinical populations. Only a few studies have suggested that the factor structure of clinical and non-clinical samples might differ [17,18]; one study compared the patient group with a student sample, not a normative sample [17]. It remains to be established if the factor structure of the TAS-20 differs between clinical and non-clinical samples.

Furthermore, disturbed family functioning and maternal alexithymia may increase the probability of alexithymia in children [19,20]. Alexithymia has certain developmental aspects which suggest that there should be age differences in alexithymic tendencies in a normal population [21]. However, only a linear correlation between alexithymia scores on the TAS-20 and age has been reported [4,6,7,22]. Heterogeneous data have been obtained. One study [4] showed a low correlation (r = -0.13, p < 0.01), while other studies [6,7,22] did not show a significant correlation. These studies would not comprehensively demonstrate a relationship between age and alexithymia if the TAS-20 scores by age have a nonlinear distribution, if the age range of the sample is limited, or if there are different developmental patterns for the three factors that make up the TAS-20. Therefore, the use of analysis of variance and multiple comparisons with a variety of different age groups is needed to clarify details of age-related differences in alexithymia. However, as far as we know this method has scarcely been used. One study [23] adopted this strategy, and showed that TAS scores were significantly greater in the higher age groups, but the age-range of the study was 21–64 years, hence the details of age effects, including teenage, on alexithymia are unknown.

Thus, the first purpose of the present study was to validate the TAS-20 with a large Japanese community sample including a wide range of ages. We examined the cross-cultural validity of the Japanese TAS-20 by use of the NEO Five-Factor Inventory (NEO-FFI) [13,14,24], a comparable personality inventory cross-culturally validated for Japanese individuals. Factor analysis was used to compare the fit of several competing factor models in a large clinical outpatient sample with psychosomatic diseases and a non-clinical sample. Our second purpose was to evaluate age-related differences in TAS-20 scores, in order to clarify the developmental aspects of alexithymia, as well as its gender-related differences. This was achieved by use of a large community sample of Japanese that included a wide range of ages, including teenagers, divided into several age groups.

## Methods

### Subjects

The community (normative) sample in this study consisted of 2,718 Japanese subjects {1,348 men and 1,370 women; age range of 14–84 years, mean age (SD) = 41.1(13.4)} residing in cities and towns in 16 prefectures throughout Japan. TAS-20 and NEO-FFI questionnaires were sent to 4,000 people, and 2,718 returned completed questionnaires (collection rate = 68.0%). These participants worked in business companies, agricultural cooperatives, fitness clubs, schools (teachers), residents' associations, universities and colleges, and the civil service. Almost all of the participants had graduated from college and were white-collar workers.

For the test-retest study, we used a sample of 196 female college students, who were requested to complete the TAS-20 and the NEO-FFI questionnaires on two occasions 11 weeks apart. Of these students, 164 completed both the test and retest answer sheets (collection rate = 83.7%; age range, 19–29 years; mean age (SD) = 20.3(1.12) years)

TAS-20 questionnaires were also collected from 1,924 patients from outpatient clinics of the Psychosomatic Departments of two large national hospital centers. The patients were diagnosed by medical doctors specializing psychosomatic medicine, with depressive disorders (n = 433, 22.5%), anxiety/phobia/panic disorders (n = 306, 15.9%), eating disorders (n = 296, 15.4%), autonomic dystonia (n = 250, 13%), gastrointestinal disorders (n = 227, 11.8%), pain and somatoform disorders (n = 163, 8.5%), headaches (n = 85, 4.4%), insomnia (n = 72, 3.7%), medical diseases (hypertension, diabetes, etc.; n = 41, 2.1%), maladjustment (n = 29, 1.5%), psychogenic reaction (n = 26, 1.4%), dermatological diseases (n = 22, 1.1%), asthma (n = 16, 0.8%), and other ailments (psychosis, gynecological problems, personality and post-traumatic stress disorders, alcohol and drug addictions, obsessive compulsive disorder, etc.; n = 40, 2.1%). This sample included 712 males and 1,212 females, mean age (SD) = 35.4(15.6) years, age range; 12–87 years.

Written informed consent was obtained from the normative subjects, and oral informed consent was given by patients visiting outpatient clinics at Kyushu University Hospital and Kohnodai Hospital at the time of a screening questionnaire given at the first visit. The study plan followed the 2002 guidelines for epidemiological surveys developed by the Japanese Ministry of Education, Culture, Science and Technology and the Ministry of Health, Labour and Welfare, was approved by our local ethics committee(18-2-Ji3) and was conducted in accordance with the Declaration of Helsinki.

### Measures and procedure

#### Translation and back translation of the TAS-20

The TAS-20 is a self-report questionnaire which consists of 20 items [4]. Each item is rated on a five-point Likert scale ranging from 1 (strongly disagree) to 5 (strongly agree), with five items negatively keyed. The TAS-20 was once previously translated into Japanese [25]; however, the back-translation method was not used resulting in several items for which the English and Japanese did not correspond. Therefore, with permission of the original author, our new translation of the TAS-20 into Japanese was carefully done using the back-translation method. The original English TAS-20 was translated in collaboration with a native English speaker who had lived in Japan for more than 12 years and who was well-acquainted with Japanese culture to insure that there were no differences in nuance between the original English and the new Japanese version. The version translated in Japanese was then back-translated into English by a person who is good at both English and Japanese to check for differences between the back-translated and original versions. The back-translated version of the TAS-20 was then sent to the author for confirmation of its accuracy [15,16].

#### NEO-FFI

Regarding the structure of personality traits, five factors have repeatedly been found to account for a large amount of the variance in the data from studies of personality, irrespective of sampling procedures, instruments used, and techniques for factor analysis [26]. The NEO-FFI is one of the standard measures of the big five factor model, and the Japanese version has been validated in the general population [13,14]. The NEO-FFI is an abridged version of the NEO-PI-R (the NEO Personality Inventory), a widely used measure designed to provide a general description of normal personality [24]. The answer format is a 5-point Likert-type scale (0–4), ranging from ''Strongly disagree'' (0) to ''Strongly agree'' (4). This scale is comprised of 60 items. The five major domains (factors) of personality measured by NEO-FFI are: Neuroticism (N), Extraversion (E), Openness to Experience (O), Agreeableness (A), and Conscientiousness (C). Scores are summed totals and have a range of 0–48 for each of the five personality domains. The Japanese version of NEO-FFI has been well cross-validated and its reliability has been confirmed [14]. High correlations (r = 0.82–0.92) between respective domains of the Japanese version of the NEO-PI-R and the NEO-FFI confirm that the two questionnaires have the same factorial structure.

#### Factorial validity with exploratory factor analysis

We conducted an exploratory factor analysis (EFA) of the TAS-20 items for both the normative and patient samples to check the consistency of the factorial structure of the Japanese TAS-20 with that of the original English version. To choose the number of factors for extraction, 'eigenvalue >1' criteria [27] and scree plot identification [28] were used, as well as the Velicer's Minimum Average Partial (MAP) Test [29] searching for the smallest average squared correlation (indicating the minimum number with a low risk of overestimation), the parallel analyses by Principal Components analysis (PA1), and the Principal Axis/common factor analysis with squared multiple correlation (PA-SMC; indicating the maximum number with a low risk of underestimation) [30].

#### Confirmatory factor analysis

In order to validate the factor structure of the Japanese version of the TAS-20 (corresponding to the three-factor model of alexithymia proposed and validated in earlier studies in English-speaking countries [4,8,9,31], we conducted maximum-likelihood confirmatory factor analysis (CFA) with the normative sample data set. The goodness-of-fit was evaluated by the following three criteria recommended by Cole and Marsh et al [32,33]: goodness-of-fit (GFI) > 0.85, adjusted goodness-of-fit (AGFI) > 0.80, and root-mean-square residual (RMSR) <0.10. However, the GFI, AGFI and RMSR are all dependent on sample size and tend to indicate a good fit in a large sample. Thus, a good fit might be obtained as an artifact of sample size, regardless of the real fit, in the present study. We also calculated the Tucker-Lewis index (TLI) [34], comparative fit index (CFI), root mean square error of approximation (RMSEA) [30], and upper and lower end of the 90% confidence interval for the RMSEA to see if the interval includes the area of "close fit" at 0.05. TLI values of 0.95 or higher are recommended. However, Schumacker and Lomax [35] contend that values close to 0.90 reflect a good model fit. The global fit indices are also supported by a RMSEA > 0.08 (preferably close model fit of < 0.06) [30] and a CFI > 0.90.

To assess the possibility of response bias to the negatively keyed items in the third factor [9], we validated a four-factor model using another CFA; i.e., the original DIF, DDF, EOT included in the three-factor model and additionally the negatively-keyed items (NKI; item 4, 5, 10, 18, and 19) added as a fourth factor. We also checked a two factor structure by CFA: the items assessing difficulty identifying feelings and difficulty describing feelings as a single factor (DIDF; DIF plus DDF), based on a model proposed in previous studies [17,18], and the items assessing externally oriented thinking as a second factor (EOT). To compare the fitness of the three-factor model with that of the four-and two-factor models, Akaike's information criterion (AIC) [36] and Bayesian Information Criterion (BIC) [37] were used. The model that yields smaller AIC and BIC values is considered more valid.

#### Reliability

Cronbach's α and mean inter-item correlation coefficients (MIC) were calculated for the total scale and for each of the three factor scales in the normative sample data set. An acceptable range of MIC for the optimal level of homogeneity was about 0.2–0.4 (proposed by Briggs and Cheek [38]). If the MIC is lower than 0.1, the single total score on a factor cannot adequately represent the complexity of the items. If it is higher than 0.5, the items on a scale tend to be redundant and the construct measured is too specific. To ensure consistency, we adopted the test-retest method to investigate the intraclass correlation coefficients (ICC) of respective factor scales for the test and retest data from the college sample. Although validation studies concerning TAS-20 often used 4 weeks or less as the test-retest interval [e.g., [4,6]], it is preferable that the consistency be validated with as long an interval as possible. We, therefore, decided on an interval of 11 weeks between the two tests.

#### Construct validity

##### Convergent validity

In a previous study [16], we examined the correlation between the Japanese version of the TAS-20 and the Structured Interview of the Beth Israel Hospital Psychosomatic Questionnaire (SIBIQ). Both measurements were developed to detect alexithymia, and a significant correlation (r = 0.49 p < 0.05) was found between the total scores of the two measures. Therefore, we concluded that the convergent validity of the TAS-20 in Japanese had been confirmed and did not require further validation.

##### Concurrent (criterion-related) validity

We calculated the correlation between the TAS-20 scores (total and each factor) and the NEO-FFI scores (each major domain) to investigate the personality pattern of alexithymia. Then we conducted a stepwise forward multiple linear regression analysis (dependent variable = TAS-20 total score, independent variables = respective factor scores of the NEO-FFI) to reveal which personality factors contributed more to alexithymia.

#### Effects of age and gender on alexithymia

In order to assess the effects of age on alexithymia, we first investigated the correlation between age and TAS-20 scores (total and each subscale) in a normal sample. We divided the sample into six groups [14–19 years old. (under 19; n = 101, 3.7%, 55 males), 20–29 y.o. (n = 540, 19.9%, 193 males), 30–39 y.o. (n = 545, 20.1%, 294 males), 40–49 y.o. (n = 733, 27%, 361 males), 50–59 y.o. (n = 597, 22% 341 males), 60–84 y.o. (over 60; n = 202, 7.4% 104 males)]. A two-way analysis of variance (age group by gender group) was done to look for any interaction of gender with age group. The mean TAS-20 scores (total and three factors) of the male and female groups were compared.

#### Software and statistical significance

The SPSS version 11.5 was used for statistical processing,. Statistical significance was set at p < 0.05. AMOS version 4.0 was also used for CFA. As a matter of convenience, we empirically describe the intensity of correlation based on the following criteria: |r | > 0.7 is strong; 0.4 < |r| < 0.7 is moderate; 0.2 < |r| < 0.4 is low or weak; and |r| < 0.2 is very weak or almost nonexistent.

## Results

### Overall psychometric properties of the TAS-20 and NEO-FFI

The respective score range and mean (SD) of each of the domains of the TAS-20 of the normative and patient samples and the NEO-FFI factors of the normative sample are shown in Table 1. We did not complement the missing values, and each factor and total score including missing items were discarded. We were able to obtain almost the same mean and SD in each factor (normative; n = 2465, patients; n = 1630: data not shown) even after excluding all the subjects with one or more missing values.

**Table 1:** TAS-20 and NEO-FFI scores in the normative sample and comparison of age and TAS scores by two-sample t-test between the normative and patient samples

	Normative					Patient							
					
	n	min	max	mean	sd	n	min	max	mean	sd	T	df	p
TAS-20													
DIF	2592	7	35	14.4	5.2	1635	7	35	19.7	6.4	t29.3	4225	<0.0001
DDF	2640	5	25	14.3	3.7	1640	5	25	15.8	4.1	t12	4278	<0.0001
EOT	2600	8	35	19.6	4	1639	8	32	19.9	3.9	t2.6	4237	<0.01
Total	2465	23	83	48.3	8.9	1630	24	88	55.4	10.4	t23.4	4093	<0.0001
													
NEO-FFI													
N	2637	4	48	25.5	7.2								
E	2596	2	44	25.4	6								
O	2577	7	47	29.4	5								
A	2560	6	48	30.6	5.2								
C	2608	5	44	27.2	4.9								

DIF = difficulty in identifying feelings, DDF = difficulty in describing feelings, EOT = externally oriented thinking, N = Neuroticism, E = Extraversion, O = Openness to Experience, A = Agreeableness, and C = Conscientiousness

### Statistical analyses

#### Factorial validity

##### Exploratory factor analysis

Table 2 shows the results of a principal component analysis with the normative and patient samples to explore the factor structure of the TAS-20. The sample performed adequately on the Kaiser-Meyer-Olkin measure (= 0.90 > minimum acceptable level = 0.50), as well as Bartlett's test of sphericity (χ^2 ^= 9948.6, df = 190, P < 0.0001). Eigenvalues of 1, 2, 3, 4, 5, and 6 number factors to be extracted were as follows: 4.53, 2.02, 1.34, 1.22, 1.03, and 0.92, respectively. If we chose 'eigenvalue >1' criteria [27], up to five factors were allowed, but the scree plot [28] identified four factors for extraction. These four components accounted for 31.9% of the total variance. MAP, PA1, and PA-SMC in the normative sample indicated the numbers of factors to be extracted as follows: 1 by MAP, 4 by PA1, 7 by PA-SMC, while the numbers in the patient sample were 1 by MAP, 4 by PA1, and 6 by PA-SMC. The preferable number of factors could be any one among 1 to 7 in the normative sample and 1 to 6 in the patient sample, but is inferred to be around 4 in both samples.

**Table 2:** Promax-rotated principal factor standardized regression coefficients of the TAS-20 and correlation coefficients between factors.

	2-factor				3-factor						4-factor								5-factor									
				
	Normative		Patient		Normative			Patient			Normative				Patient				Normative					Patient				
								
Items	I	II	I	II	I	II	III	I	II	III	I	II	III	IV	I	II	III	IV	I	II	III	IV	V	I	II	III	IV	V
Standardized Coefficients																												
*DIF*																												
1	**0.69**	0.00	**0.72**	-0.03	**0.61**	0.11	0.00	**0.60**	0.17	-0.03	**0.61**	0.15	0.04	-0.06	**0.54**	0.25	-0.02	-0.01	**0.58**	0.18	0.00	0.04	-0.10	**0.73**	-0.02	0.09	-0.04	-0.08
3	**0.45**	-0.08	**0.45**	-0.05	**0.53**	-0.07	-0.06	**0.60**	-0.16	0.05	**0.52**	-0.04	-0.02	-0.07	**0.59**	-0.13	0.04	-0.02	**0.55**	-0.03	0.06	-0.16	0.03	-0.17	**0.73**	0.02	0.08	0.06
6	**0.63**	-0.05	**0.68**	-0.03	**0.64**	0.01	-0.04	**0.63**	0.08	0.00	**0.63**	0.00	-0.06	0.05	**0.58**	0.16	0.01	-0.02	**0.61**	0.01	-0.07	0.04	0.00	**0.70**	0.04	0.01	-0.01	-0.09
7	**0.64**	-0.03	**0.57**	-0.20	**0.82**	-0.18	0.00	**0.81**	-0.25	-0.08	**0.81**	-0.16	0.01	-0.01	**0.82**	-0.22	-0.09	-0.04	**0.84**	-0.16	0.08	-0.10	0.04	0.11	**0.70**	-0.12	-0.08	0.01
9	**0.68**	-0.06	**0.7**	-0.12	**0.68**	0.02	-0.05	**0.68**	0.05	-0.08	**0.67**	0.03	-0.05	0.00	**0.65**	0.09	-0.10	0.02	**0.65**	0.04	-0.07	0.05	-0.04	0.33	**0.36**	0.10	-0.09	0.03
13	**0.74**	0.06	**0.66**	-0.02	**0.65**	0.12	0.07	**0.77**	-0.09	0.08	**0.64**	0.11	0.04	0.07	**0.76**	-0.07	0.05	0.03	**0.64**	0.11	0.05	0.02	0.07	0.34	**0.45**	-0.04	0.05	0.04
14	**0.57**	-0.01	**0.59**	0.07	**0.57**	0.01	0.00	**0.46**	0.18	0.06	**0.57**	-0.01	-0.03	0.06	**0.43**	0.18	0.02	0.09	**0.55**	0.00	-0.04	0.07	0.00	**0.72**	-0.11	0.01	-0.01	0.02
*DDF*																												
2	**0.57**	-0.02	**0.68**	0.03	0.21	**0.47**	-0.05	**0.45**	0.30	-0.02	0.20	**0.53**	0.00	-0.09	0.34	**0.44**	0.03	-0.08	0.17	**0.56**	-0.04	0.02	-0.10	0.21	0.17	**0.43**	0.04	-0.06
4*	**0.39**	0.12	**0.45**	0.13	0.08	**0.39**	0.10	0.23	**0.29**	0.08	0.05	**0.54**	0.23	-0.20	0.07	**0.50**	0.18	-0.18	0.03	**0.58**	0.18	-0.03	-0.15	0.05	0.03	**0.51**	0.19	-0.15
11	**0.46**	-0.03	**0.53**	0.12	-0.04	**0.65**	-0.08	0.15	**0.49**	-0.01	-0.04	**0.61**	-0.13	0.08	0.09	**0.50**	-0.04	0.13	-0.03	**0.57**	-0.12	0.01	0.11	0.07	0.05	**0.49**	-0.03	0.16
12	**0.38**	0.06	**0.40**	0.12	-0.03	**0.52**	0.03	-0.02	**0.53**	-0.03	-0.04	**0.47**	-0.06	0.15	-0.08	**0.55**	-0.04	0.10	-0.02	**0.43**	-0.04	0.03	0.18	0.11	-0.14	**0.49**	-0.04	0.11
17	**0.27**	0.01	**0.34**	0.07	-0.06	**0.41**	-0.02	-0.04	**0.48**	-0.07	-0.08	**0.36**	-0.12	0.18	-0.10	**0.50**	-0.07	0.09	-0.04	0.31	-0.02	-0.11	**0.33**	-0.09	-0.03	**0.52**	-0.06	0.13
*EOT*																												
5*	0.00	**0.45**	-0.06	**0.48**	-0.09	0.09	**0.45**	-0.10	0.04	**0.47**	-0.11	0.04	**0.32**	0.25	-0.08	-0.05	**0.39**	0.18	-0.19	0.05	0.12	**0.51**	-0.03	0.09	-0.13	-0.09	**0.39**	0.15
10*	-0.01	**0.50**	-0.02	**0.45**	0.04	-0.09	**0.51**	0.06	-0.12	**0.53**	0.04	-0.04	**0.55**	0.00	0.03	-0.09	**0.53**	0.00	0.05	0.00	**0.49**	0.10	-0.04	-0.02	0.05	-0.06	**0.53**	-0.01
18*	-0.10	**0.38**	0.04	0.25	-0.02	-0.12	**0.39**	-0.04	0.10	**0.23**	-0.01	-0.08	**0.43**	-0.03	-0.12	0.20	**0.30**	-0.11	0.03	-0.05	**0.47**	-0.04	0.01	-0.11	-0.03	0.23	**0.31**	-0.09
19*	-0.07	**0.63**	0.01	**0.49**	-0.09	0.00	**0.62**	0.05	-0.06	**0.55**	-0.09	0.03	**0.63**	0.06	0.00	-0.01	**0.60**	-0.04	-0.04	0.06	**0.70**	0.01	0.10	-0.07	0.06	0.04	**0.61**	-0.04
8	0.31	**0.36**	0.21	**0.43**	0.21	0.12	**0.36**	0.07	0.17	**0.39**	0.19	0.01	0.16	**0.40**	0.13	-0.02	0.24	**0.40**	0.12	-0.04	-0.05	**0.59**	0.11	0.23	-0.02	-0.07	0.24	**0.37**
15	**0.23**	0.12	0.19	0.20	0.09	**0.17**	0.11	-0.09	**0.34**	0.09	0.07	0.09	-0.04	**0.29**	-0.06	0.19	-0.08	**0.43**	0.12	0.02	0.03	0.02	**0.33**	0.00	-0.03	0.17	-0.07	**0.43**
16	0.04	0.16	-0.01	0.16	-0.09	**0.15**	0.14	-0.18	**0.20**	0.08	-0.11	0.06	-0.02	**0.30**	-0.12	0.04	-0.06	**0.35**	-0.07	0.00	0.04	0.06	**0.31**	-0.19	0.03	0.08	-0.06	**0.38**
20	0.12	**0.26**	0.19	0.19	0.06	0.06	**0.26**	0.08	0.14	**0.16**	0.05	-0.05	0.08	**0.36**	0.14	-0.01	0.02	**0.32**	0.06	-0.09	0.07	0.20	**0.25**	-0.07	0.19	0.04	0.04	**0.35**

Correlations between factors																												
I		-0.04		-0.13		0.64	-0.01		0.61	-0.13		0.63	-0.07	0.23		0.66	-0.09	0.09		0.61	-0.19	0.25	0.21		0.71	0.65	-0.05	0.17
II							0.02			0.04			-0.02	0.20			0.01	0.12			-0.13	0.22	0.23			0.53	-0.18	-0.04
III														0.26				0.25				0.35	0.00				-0.06	0.06
IV																							0.23					0.26

Note; Items 4,5,10,18 and 19 are negatively keyed (*). Bold type in each item shows the largest coefficient among three or five factors. DIF = difficulty in identifying feelings, DDF = difficulty in describing feelings, EOT = externally oriented thinking

After promax rotation, a principle component analysis with 3 factors for extraction showed that almost all items had salient standardized regression coefficients for one of the three factors (Table 2). A pattern matrix almost identical to the original three-factor model emerged. Two items (No. 15; "I prefer talking to people about their daily activities rather than their feelings", and No. 16; "I prefer to watch "light" entertainment shows rather than psychological dramas") did not belong to the same factor (EOT) as in the original model and showed low coefficients.

To elucidate the factors' contents further (especially EOT which had relatively low internal reliability), as shown in Table 2, we also conducted principal component analyses with the four and five factors for extraction, because the number of factors allowed to be extracted is up to five according to the 'eigenvalue > 1' criteria (see above). The first and second extracted factors are almost the same as the respective factors in the original three-factor model. However, in the 4-factor extraction the original EOT was divided into two factors (III and IV) of positively and negatively keyed items.

The normative and patient samples showed similar extracted factors in each 3- and 4-factor extraction, although the factor loadings in two groups differed in each 2- and 5-factor extraction.

##### Confirmatory factor analysis

CFA was done with the normative and patient sample data sets for each 2-, 3-, and 4-factor solution model. All the standardized parameter estimates are shown in Table 3, and the estimates of covariance and correlations between factors in each model are shown in Table 4. In the 3-factor solution model, the correlation between DIF and DDF was moderate, but EOT had a relatively weak correlation with the other factors.

**Table 3:** Standardized parameter estimates by confirmatory factor analysis of the TAS-20: Estimates of standardized regression weights

	2-factor				3-factor						4-factor							
	
	Normative		Patient		Normative				Patient			Normative				Patient		
	
item No.	DIDF	EOT	DIDF	EOT	DIF	DDF	EOT	DIF	DDF	EOT	DIF	DDF	EOT	NKI	DIF	DDF	EOT	NKI
1	0.70		0.74		0.70			0.74			0.69				0.74			
3	0.47		0.46		0.47			0.46			0.47				0.46			
6	0.64		0.70		0.65			0.71			0.65				0.70			
7	0.65		0.60		0.67			0.62			0.67				0.62			
9	0.68		0.72		0.69			0.72			0.69				0.72			
13	0.74		0.67		0.74			0.68			0.74				0.68			
14	0.58		0.59		0.59			0.60			0.59				0.60			
																		
2	0.56		0.67			0.68			0.75			0.67				0.73		
4	0.38		0.44			0.50			0.52			0.63		0.30		0.63		0.30
11	0.44		0.49			0.58			0.56			0.58				0.57		
12	0.36		0.36			0.45			0.44			0.45				0.45		
17	0.24		0.31			0.32			0.38			0.33				0.38		
																		
5		0.41		0.48			0.41			0.48			0.47	0.31			0.53	0.39
8		0.30		0.40			0.30			0.40			0.67				0.65	
10		0.51		0.49			0.51			0.49			0.41	0.51			0.48	0.56
15		0.11		0.13			0.11			0.14			0.28				0.31	
16		0.14		0.12			0.14			0.13			0.18				0.18	
18		0.42		0.25			0.42			0.26			0.25	0.50			0.21	0.33
19		0.66		0.53			0.66			0.53			0.50	0.69			0.50	0.61
20		0.24		0.17			0.24			0.16			0.33				0.31	

All parameter estimates are statistically significant (p ~ 0). DIF = difficulty in identifying feelings, DDF = difficulty in describing feelings, EOT = externally oriented thinking

**Table 4:** Between-factor correlations in each model

			Normative					Patient				
			
			Estimate of correlation	Estimate of covariance	Standard error of covariance	Critical ratio for covariance		Estimate of correlation	Estimate of covariance	Standard error of covariance	Critical ratio for covariance	
2-factor												
DIDF	↔	EOT	-0.035	-0.011	0.009	-1.3	ns	-0.048	-0.024	0.018	-1.4	ns
3-factor												
DIF	↔	EOT	-0.041	-0.013	0.009	-1.5	ns	-0.077	-0.039	0.019	-2.1	*
DDF	↔	EOT	-0.011	-0.002	0.005	-0.4	ns	0.041	0.011	0.01	1.0	ns
DIF	↔	DDF	0.709	0.203	0.017	12.0	*	0.804	0.404	0.034	11.8	*
4-factor												
DIF	↔	DDF	0.722	0.167	0.014	11.9	*	0.81	0.32	0.028	11.3	*
DIF	↔	NKI	-0.426	-0.082	0.013	-6.5	*	-0.368	-0.12	0.021	-5.7	*
DIF	↔	EOT	0.388	0.151	0.013	11.4	*	0.221	0.117	0.021	5.6	*
EOT	↔	DDF	0.353	0.092	0.011	8.2	*	0.269	0.094	0.017	5.7	*
NKI	↔	EOT	-0.378	-0.082	0.024	-3.4	*	-0.484	-0.141	0.04	-3.5	*
NKI	↔	DDF	-0.419	-0.054	0.009	-5.8	*	-0.323	-0.071	0.016	-4.5	*

*p < 0.001 (two tailed). DIF = difficulty in identifying feelings, DDF = difficulty in describing feelings, EOT = externally oriented thinking

The parameters of goodness-of-fit for the conventional three-factor model and 2- and 4-factor models are shown in Table 5. The chi-square goodness-of-fit in this study seems to reflect the large sample size. The RMSR, GFI and AGFI in the conventional 3-factor model met the criteria recommended by Cole and Marsh [32,33], however we cannot deny that the high values of the GFI and AGFI are artifacts of the large sample size. RMSEA (0.061) also shows a good fit for this model, although TLI (0.82) and CFI (0.85) were not satisfactory.

**Table 5:** Parameters of goodness of fit in each CFA solution

	Normative			Patient		
	
	2-factor	3-factor	4-factor	2-factor	3-factor	4-factor
RMSR	0.080	0.073	0.046	0.093	0.086	0.070
GFI	0.915	0.933	0.956	0.912	0.926	0.942
AGFI	0.894	0.915	0.942	0.891	0.907	0.923
TLI	0.778	0.824	0.885	0.796	0.827	0.864
CFI	0.802	0.845	0.904	0.819	0.848	0.886
RMSEA	0.068	0.061	0.049	0.067	0.062	0.055
HI 90	0.071	0.063	0.052	0.070	0.065	0.058
LO 90	0.066	0.058	0.046	0.064	0.058	0.052
χ^2^	2,105.1	1,684.9	1,100.7	1,405.1	1,201.1	937.7
df	169	167	159	169	167	159
χ^2^/df	12.5	10.1	6.9	8.3	7.2	5.9
AIC	2,187.1	1,770.9	1,202.7	1,487.1	1,287.1	1,039.70
BIC	2,425.3	2,020.7	1,499.0	1,708.2	1,519.0	1,314.8

all χ^2;^; significant p ~ 0

RMSR = root-mean-square residual, GFI = goodness-of-fit, AGFI = adjusted goodness-of-fit, TLI = Tucker-Lewis index, CFI = comparative fit index, RMSEA = root mean square error of approximation, HI 90/LO 90 = upper/lower end of the 90% confidence interval for the RMSEA, AIC = Akaike's information criterion, BIC = Bayesian Information Criterion

The CFA of the four-factor model showed better fittings than the 3-factor model, and the RMSEA (0.049), TLI (0.89) and CFI (0.90) of this model are all satisfactory. The AIC and BIC of the four-factor model (1202.7, 1449.0) was better than that of the three-factor model (1770.9, 2020.7). On the other hand, the two-factor model (i.e., conventional DIF + DDF taken as one factor and EOT) showed poor goodness-of-fit, worse than the three-factor model, as indicated by the AIC and BIC scores of 2187.1 and 2425.3.

The tendency of factor loadings, between-factor correlations, and the tendency of model-fitting in the normative and patient samples were similar.

#### Reliability

Cronbach's alpha (α) for each of the three factor scales and the total scale of the TAS-20 in the normative sample data set were as follows: DIF: 0.83; DDF: 0.64; EOT: 0.54; Total: 0.75. MIC was DIF: 0.41, DDF: 0.26, EOT: 0.13, Total: 0.13. All correlations were statistically significant (p < 0.0001). The internal reliability (α) of the DIF, DDF and TAS-20 totals were acceptable, but the reliability of EOT was relatively low. The MIC of DIF and DDF were acceptable but the EOT and Total were relatively low. On the other hand, Cronbach's alpha (α) for each of the three factor scales and the total scale of the TAS-20 in the patients' sample data set were as follows: DIF: 0.84; DDF: 0.67; EOT: 0.49; Total: 0.77. MIC was DIF: 0.42, DDF: 0.29, EOT: 0.11, Total: 0.15. All correlations were statistically significant (p < 0.0001). The results for the patient sample were very similar to the normative sample, except that the alpha of EOT was worse.

In the test-retest group of college students, the coefficients of correlation between the test and retest scores of TAS-20 were as follows; DIF: 0.56; DDF: 0.67; EOT: 0.58; Total: 0.61, and all of them were statistically significant (p < 0.0001). These results indicated moderate correlation (r~ 0.6) in each factor and total TAS-20 scale. The intraclass correlation coefficients {ICC(2,1)} of therespective factor scales of the test and retest groups were as follows: DIF: 0.56; DDF: 0.67; EOT: 0.57; Total: 0.61. All the correlations were statistically significant (p < 0.0001). The test-retest showed moderate reliability for the TAS-20 total and the three factors.

#### Concurrent (criterion-related) validity of TAS-20 with NEO-FFI

The correlation coefficients were calculated for the TAS-20 (total and each factor) and NEO-FFI scores (each major domain) and are given in Table 6. As a general trend, (N) of the NEO-FFI correlated positively with TAS-20 total, DIF, and DDF. Scores for (E), (O), (A), and (C) correlated negatively with those for TAS-20 total, DIF, DDF, and EOT. EOT were negatively correlated with all NEO-FFI domains.

**Table 6:** Correlation coefficients between TAS-20 scores (total and each factor) and NEO-FFI scores

	N	E	O	A	C
DIF	0.52**	-0.17**	0.03	-0.28**	-0.24**
DDF	0.38**	-0.38**	-0.06*	-0.16**	-0.24**
EOT	-0.06*	-0.08**	-0.48**	-0.16* *	-0.16**
Total	0.43**	-0.29**	-0.21**	-0.30**	-0.31**

statistical significance: <0.005, <0.0001 DIF = difficulty in identifying feelings, DDF = difficulty in describing feelings, EOT = externally oriented thinking, N = Neuroticism, E = Extraversion, O = Openness to Experience, A = Agreeableness, and C = Conscientiousness

In the stepwise forward multiple linear regression analysis (dependent variable = TAS-20 total score; independent variable = each factor score of NEO-FFI), five independent variables were selected (contribution; R^2^= 0.309, adjusted R^2^= 0.307), and this model was statistically significant for regression (S^2^= 53117.7, df = 5, mean square = 10623.5, F = 194.9, p < 10^-171^). The standardized beta of each variable was as follows: (N): 0.365; (O): -0.198; (C): -0.169; (A): -0.106; (E): -0.074. All were significant at p < 0.0005.

#### Effects of age on the TAS-20

In investigating the effect of age on alexithymia, we first calculated the correlation coefficients between age and TAS-20 scores in the normative population data set (total and each subscale): DIF, -0.139; DDF, -0.120; EOT, 0.166; Total, -0.062 (all significant at p < 10^-9 ^for DIF, DDF, EOT; and p < 0.005 for Total). These are significant, but poor correlations.

**Figure 1:**
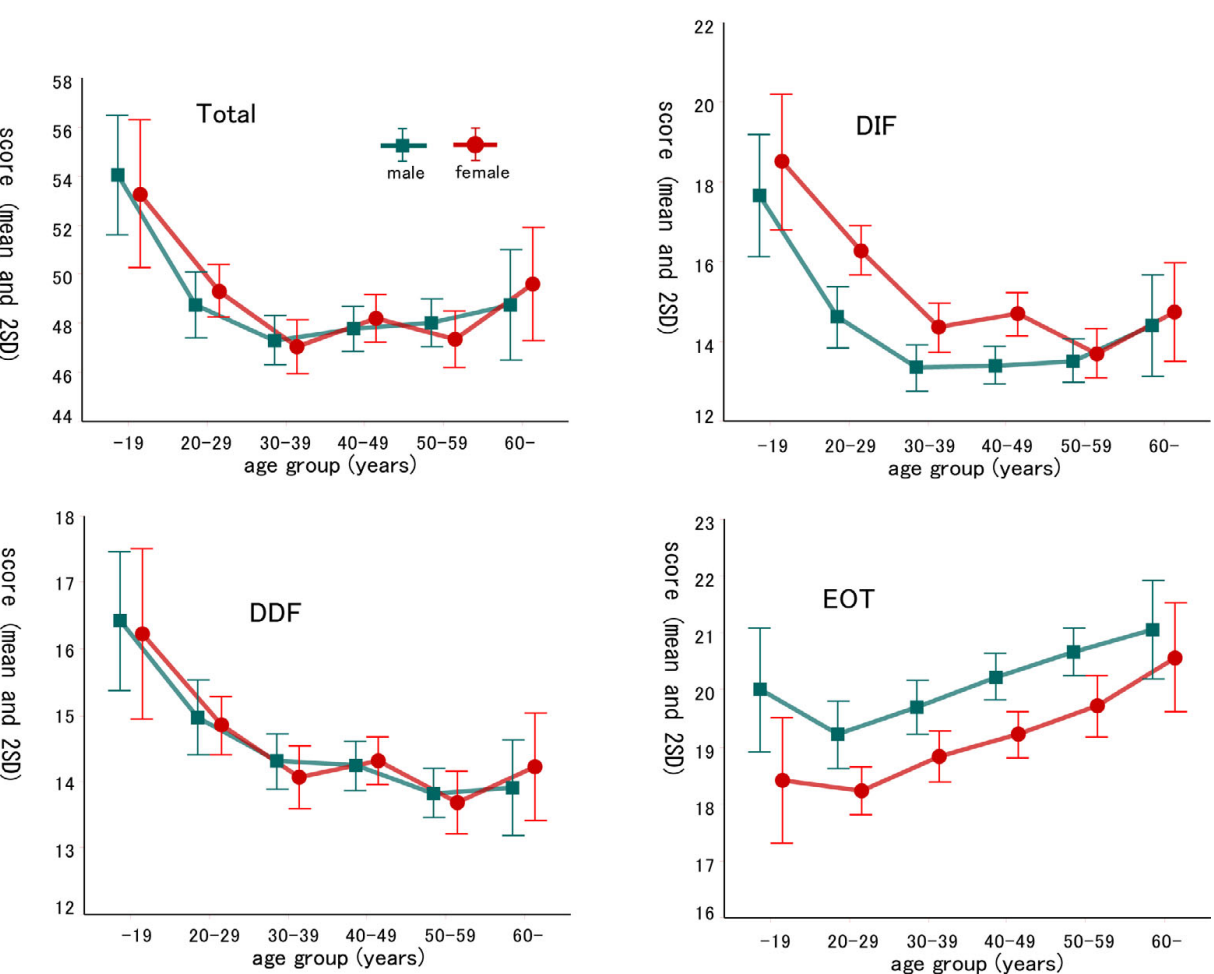
Male and female scores (mean ± SE) of TAS-20 total and each factor individually by age group. Significant differences (p < 0.05) of scores among age groups using Tukey's multiple comparison were as follows: TAS-20 total: 10s > 20s > (30s, 40s, 50s, over60), Factor 1 (DIF): 10s > (20s, 30s, 40s, 50s, over60) and 20s > (30s, 40s, 50s), Factor 2 (DDF): 10s > (20s, 30s, 40s, 50s, over60) and 20s > (30s, 40s, 50s), Factor 3 (EOT): 20s < (30s, 40s, 50s, over60), 30s < (50s, over60) and (10s, 40s) < over60.

No significant interaction was found between 'age group' and gender for any factor or total score. Figure 1 shows the mean (± SE) scores for TAS-20 Total, DIF, DDF and EOT for each of the six age groups. Table 7 shows the results of a two-way analysis of variance with gender and age group factors. Significant differences in the TAS scores (all factors and total) were found between the six age groups. Tukey's multiple comparisons between all possible pairs in the six groups showed that scores for the three factors and the total of TAS-20 were significantly different for the age groups (see Figure 1). The TAS-20 total, DIF and DDF scores are high for teenagers, but decrease with age. In particular, from age 30 the scores did not change significantly. On the other hand, EOT is clearly different from the other factors. There was an almost linear positive correlation between age and the EOT scores.

**Table 7:** Effects of gender and age on TAS scores: Two-way ANOVA

		scores in each gender group								
							
		male		female						
							
		mean	SD	mean	SD	S^2^	df	mean square	F	p
TAS										
total	gender(G)	48.2	8.7	48.4	9.1	0.34	1	0.34	0	ns
	age group(A)					4021.4	5	804.3	10.35	<10^-9^
	(G)*(A) interaction					179.4	5	35.9	0.46	ns
										
DIF	(G)	13.8	5.0	15.0	5.3	309.4	1	309.4	12.01	<0.001
	(A)					2467.3	5	493.5	19.16	<10^-18^
	(G)*(A)					179.1	5	35.8	1.39	ns
										
DDF	(G)	14.3	3.6	14.4	3.8	1.11	1	1.1	0.08	ns
	(A)					765.8	5	153.2	11.59	<10^-10^
	(G)*(A)					15.8	5	3.2	0.24	ns
										
EOT	(G)	20.1	3.9	19.0	3.9	376.3	1	376.3	24.89	<10^-6^
	(A)					905.6	5	181.1	11.98	<10^-10^
	(G)*(A)					21.8	5	4.4	0.29	ns

DIF = difficulty in identifying feelings, DDF = difficulty in describing feelings, EOT = externally oriented thinking.

#### Effects of gender on the TAS-20

The TAS-20 total and the three factor scores were compared by gender in the normative population data set (Table 7, Figure 1). Both the male and female groups showed the same effect of age on the TAS-20 total and each factor score (i.e., no significant age-group by gender interaction). DIF scores for females and EOT scores for males were significantly higher than those of the other respective gender group. No significant difference was found between the male and female groups in total and DDF scores on the TAS-20.

## Discussion

### Cross-validation of the Japanese TAS-20

In the present study, we validated a newly developed Japanese version of the TAS-20 with a large community sample that included people of a wide range of ages. The overall three-factor structure of the original English TAS-20 (DIF, DDF, and EOT) was validated, and there was also support for other models. There were some problematic issues for Japanese subjects (as for other populations) in the EOT factor because of low internal consistency due to its contextual complexity, polysemy, and negatively keyed items (NKI). The results indicate that the four-factor solution with the additional NKI factor is superior to the original three-factor model. The Total TAS-20 score has sufficient internal consistency for both the normative and outpatient groups, which demonstrates the usefulness of the Japanese version for clinical purposes. The result of relationship between the TAS-20 and the NEO five factor personality model in the present study, also confirmed in previous reports [3,39-42], supports the distinctive trait of alexithymia. Based on these findings, we endorse the TAS-20 questionnaire as a practical, useful tool for identifying people with alexithymia in Japan, with some problems remaining to be solved.

### Validity of the three-factor model of TAS-20

The EFA revealed that the number of factors to be extracted is around 4, including the original 3 factors. Most of the appropriate fit indices and the factor loadings yielded by the CFA suggested that a 4-factor model with NKI added showed a better fit than the 3-factor model. Although the items of NKI were also included in EOT, NKI showed low correlation with EOT, similar to the other two factors (see Table 4), indicating NKI in EOT to be rather independent factors. Taken together with the factor loadings of EFA and the relatively low internal consistency of EOT, NKI affected the fit of the 3-factor model. However, the 4-factor model that we created included the original DIF, DDF, and EOT factors. Because of the interpretability of these factors' contents, the three-factor model is preferable. Merely dividing the EOT into 2 different factors (positively and negatively keyed items) would reduce the interpretability of these factors. Parameter estimates and correlations among the three factors indicate that DIF and DDF correlate moderately with each other and EOT correlates weakly with the other two factors. These results are consistent with previous research [4,6,7,43], indicating that the ability to communicate feelings to others is related to one's ability to recognize one's own emotions. An externally-orientated cognitive style contains little reference to a person's inner feelings [4,6,7,43]. Although the two factor solution (i.e., DIF+DDF and EOT) was proposed in some previous reports [44,45], the result of our CFA shows a poorer fit and suggests that identifying and describing inner feelings are different. DIF and DDF should remain separate from each other [9].

The EOT of the TAS-20 showed relatively low reliability, although the TAS-20 total, DIF and DDF had acceptable levels of reliability. This is consistent with findings in France, Austria, Italy, Portugal, the Netherlands, Lithuania, Peru, Poland, South Korea, and Taiwan [10]. Taken together with the result of low MIC for EOT, it is partly because the third factor, 'Externally oriented thinking', contains various components. Furthermore, EOT contains more negatively keyed items than the other two factors [10], contributing to the complexity of EOT. This was supported by the better fit of the four-factor model of TAS-20 by CFA with the additional factor composed of those negatively keyed items.

The normative and patient samples showed similar extracted factors in each 3- and 4-factor extraction, while there were different extracted factors in each 2- and 5-factor extraction. Hence, we also recommend the 3- or 4-factor solution for clinical use.

The test-retest validation shows moderate and significant correlations between the test and retest measurement scores for each factor and the total TAS-20 (~ 0.6). However these are not as strong as the correlations in other studies of the TAS-20 [4,6,7]. This discrepancy may be explained by some state-dependent dimension (like depression [46,47]) or to the relatively small standard deviation of the TAS-20 score (test: 8.5, retest: 8.7), which is perhaps related to the homogenous educational status in the present sample. Another explanation is that the interval period between test and restest adopted in this study was almost three months, longer than that in other validation studies [e.g., [4,6]]. Taking into account these considerations, the TAS-20 scales should be considered sufficiently consistent.

### Concurrent validity of TAS-20 as a personality trait

Our examination of concurrent (criterion-related) validity showed a significant moderate positive correlation of the TAS-20 total score with Neuroticism of the NEO-FFI and weak but significant negative correlations with the other domains of the NEO-FFI. These findings are consistent with previous studies [3,39-42]. The beta values demonstrate that high Neuroticism is the greatest explanatory factor related to alexithymia, with low Openness and low Conscientiousness also contributing. People with high Neuroticism tend to think unrealistically, to be unable to control their anger, and to be poor at coping with stressors [24]. Poor coping and use of repression as a coping style was also reported in alexithymic people in a previous study [48]. Specifically, there was a significant moderate negative correlation between EOT and Openness which coincides with the result of a previous study [3]. People with low Openness are deficient in imaginative activity, and tend not to seek out new experiences, and therefore may be limited in their opportunities to describe their emotions to others or learn about others' descriptions of emotions. The salient correlation between Openness and EOT supports that EOT would correspond to a passive and negative attitude toward observing, analyzing, and coping with unknown events and conflicts in one's mind. This is compatible with the finding that people with alexithymia have negative attitudes and are poor at coping with stressors [48]. Similarly, individuals low in Conscientiousness are associated with lack of self-control and consistent effort [24]. These salient personality dimensions are consistent with the characteristics of alexithymia, indicating that alexithymia may be a specific type of personality trait. We suspect that negative attitude is one of the defining aspects of alexithymia. Poor coping with stressors among people with alexithymia might be related to their high risk of psychiatric and psychosomatic diseases.

### Age-related differences and developmental aspects of alexithymia

Multiple comparisons between the six age groups demonstrated a developmental aspect of alexithymia. The DIF, DDF and Total TAS-20 scores of teenagers were relatively higher than those people in their 20s and 30s. This trend was consistent for males and females. One previous study [4] that included a correlation analysis between age and TAS scores showed a low correlation (r = -0.13, p < 0.01), while other studies [6,7,22] did not show a significant correlation. However, alexithymia is suspected to be associated with developmental issues. Taylor et al. [2] described how the cognitive ability to recognize and control one's emotion is acquired during development and that failure to acquire it might be connected with alexithymia. An association between the development of speech in early childhood and alexithymia 30 years later has also been described [49]. It has been reported that disturbed family functioning and maternal alexithymia increase the probability of alexithymia in children [19,20]. Therefore we should consider that self-awareness ability, the lack of which constitutes alexithymia, could be obtained step-by-step in developmental stages. Close and mature relationships and sharing with others occur in the next adulthood stage on the basis of an established individual identity. We presume that younger people in adolescence are cognitively less capable of looking into their inner emotional states, and identifying and/or describing them. Our results suggest that personal insight seems to be acquired with maturity, and reaches its full potential in one's thirties. This is consistent with the theory of Erikson's stages of psychosocial development [50], which proposed that acquiring an individual identity continues during later adolescence (twenties), and early adulthood. The twenties are supposed to be a developmental period of acquiring cognitive ability through various experiences.

A limitation of the present study is that the study design is cross-sectional. When we are inferring age-related effects on alexithymia, there is another possible explanation that the results of age-related difference of TAS-20 scores might only reflect the tendency that young people have become poorer at recognizing and describing their emotions than in the past. A follow-up study will be necessary to clarify this point.

Externally oriented thinking (EOT), on the other hand, showed different distributions of scores by age group (i.e., linear increase with age). Bagby et al reported almost the same result [3]. This factor showed a lower correlation with the other two factors, suggesting that EOT has a considerably different character from the other two factors of the TAS-20. EOT has an aspect of negativity and inability to cope with stressors (as described above) rather than of inner feeling. Therefore, the results might reflect that, as people get older, they lose interest in seeking out novelty as they did in their youth. McCrae reported that there are significant cross-sectional declines with age in Openness in Spanish, Czech, and Turkish samples [11]. This finding is in line with our present observation of a high correlation of EOT with Openness and age-related distribution of EOT (in our cross-validation report). Considering these synchronized age-related differences of both the TAS-20 and NEO scales, such a cross-cultural similarity in the relationship between NEO factors and age makes us think that the different TAS-20 scores related to different age groups in our present study reflect developmental aspects of alexithymia, although we should not overlook potential sampling bias or social trends between different countries. In addition, we propose that an alexithymia scale for younger people is worth developing, as has already happened with the NEO-FFI [14,24].

### Gender and alexithymia

The investigation of the effect of gender on alexithymia revealed a significantly higher DIF in females and EOT in males. These findings mean that females are not as good at identifying inner emotions as males, and males tend to be more externally-oriented in their thinking. There was no gender difference in DDF scores. These gender differences on these two factors resulted in no gender difference in the total scores. This finding is partly consistent with a cross-cultural study that Asian males showed higher EOT scores than European American males [51]. Additional cross-cultural studies are needed to clarify possible cultural influences on gender difference of alexithymic tendencies.

## Conclusion

Our newly developed Japanese version of the TAS-20 was validated with a large, community sample. Although the four-factor model with the additional NKI factor was found to be superior to the original three-factor model, we concluded that the factors (DIF, DDF, and EOT) of the original TAS-20 were generally supported and useful because of their interpretability. We found limitations in the reliability of EOT, as has also been found in many other studies. Changing some EOT items, including re-evaluating the negative keyed items and reducing polysemy, might improve the reliability and the model-fitting. The similar factor structure of the clinical and normative samples indicates that the new Japanese version of the TAS-20 is appropriate for clinical use. The factor analysis and correlation with the NEO-FFI provided cross-validation, and suggested that alexithymia includes the characteristic personality trait; high neuroticism combined with low openness to experience and low conscientiousness. The evident age-related differences in the TAS-20 scores suggest that there is a developmental aspect associated with the features of alexithymia, characteristic of each factor on the TAS-20, such that younger people should be evaluated separately from older people. Younger people are presumed to be cognitively less capable of looking into their inner emotional states and of identifying and/or describing them. The linear increase with age of EOT scores may reflect that people lose openness or interest in novelty as they get older, and suggests that EOT has a considerably different character from the other two factors of the TAS-20.

## Authors' contributions

YM performed the statistical analysis and drafted the manuscript. MM, MS and T. Igarashi participated in the design of the study and collected the normative sample data. T. Ishikawa and CK collected the clinical data set. YM, MM and GK conceived of the study and participated in its design. All authors read and approved the final manuscript.

## References

[B1] Sifneos PE (1967). Clinical observations on some patients suffering from a variety of psychosomatic diseases. Acta Medicina Psychosomatica.

[B2] Taylor GJ, Bagby RM, Parker JDA (1997). Disorders of affect regulation: Alexithymia in medical and psychiatric illness.

[B3] Bagby RM, Taylor GJ, Parker JD (1994). The Twenty-item Toronto Alexithymia Scale-II. Convergent, discriminant, and concurrent validity. J Psychosom Res.

[B4] Bagby RM, Parker JD, Taylor GJ (1994). The twenty-item Toronto Alexithymia Scale-I. Item selection and cross-validation of the factor structure. J Psychosom Res.

[B5] Simonsson-Sarnecki M, Lundh LG, Torestad B, Bagby RM, Taylor GJ, Parker JD (2000). A Swedish translation of the 20-item Toronto Alexithymia Scale: cross-validation of the factor structure. Scand J Psychol.

[B6] Bressi C, Taylor G, Parker J, Bressi S, Brambilla V, Aguglia E, Allegranti I, Bongiorno A, Giberti F, Bucca M, Todarello O, Callegari C, Vender S, Gala C, Invernizzi G (1996). Cross validation of the factor structure of the 20-item Toronto Alexithymia Scale: an Italian multicenter study. J Psychosom Res.

[B7] Pandey R, Mandal MK, Taylor GJ, Parker JD (1996). Cross-cultural alexithymia: development and validation of a Hindi translation of the 20-Item Toronto Alexithymia Scale. J Clin Psychol.

[B8] Bagby RM, Taylor GJ, Parker JD, Loiselle C (1990). Cross-validation of the factor structure of the Toronto Alexithymia Scale. J Psychosom Res.

[B9] Parker JD, Taylor GJ, Bagby RM (2003). The 20-Item Toronto Alexithymia Scale. III. Reliability and factorial validity in a community population. J Psychosom Res.

[B10] Taylor GJ, Bagby RM, Parker JD (2003). The 20-Item Toronto Alexithymia Scale. IV. Reliability and factorial validity in different languages and cultures. J Psychosom Res.

[B11] McCrae RR, Costa PT, Ostendorf F, Angleitner A, Hrebickova M, Avia MD, Sanz J, Sanchez-Bernardos ML, Kusdil ME, Woodfield R, Saunders PR, Smith PB (2000). Nature over nurture: temperament, personality, and life span development. J Pers Soc Psychol.

[B12] Costa PT, Terracciano A, McCrae RR (2001). Gender differences in personality traits across cultures: robust and surprising findings. J Pers Soc Psychol.

[B13] Shimonaka J (1996). Report on the Japanese version of the NEO PI-R.

[B14] Shimonaka J, Nakazato K, Gondo K, Takayama M (1999). NEO-PI-R, NEO-FFI common manual (for Adults and college studens).

[B15] Komaki G, Maeda M, Arimura T, Nakata A, Shinoda H, Ogata I, Shimura M, Kawamura N, Kubo C (2003). The reliability and factorial validity of the Japanese version of the 20-item Toronto Alexithymia Scale [abstract]. J Psychosom Res.

[B16] Arimura T, Komaki G, Murakami S, Tamagawa K, Nishikata H, Kawai K, Nozaki T, Takii M, Kubo C (2002). Development of the Structured Interview by the modified edition of Beth Israel Hospital Psychosomatic Questionnaire (SIBIQ) in Japanese Edition to evaluate alexithymia. Jpn J Psychosom Med.

[B17] Kooiman CG, Spinhoven P, Trijsburg RW (2002). The assessment of alexithymia: a critical review of the literature and a psychometric study of the Toronto Alexithymia Scale-20. J Psychosom Res.

[B18] Muller J, Buhner M, Ellgring H (2003). Is there a reliable factorial structure in the 20-item Toronto Alexithymia Scale? A comparison of factor models in clinical and normal adult samples. J Psychosom Res.

[B19] Lumley MA, Mader C, Gramzow J, Papineau K (1996). Family factors related to alexithymia characteristics. Psychosom Med.

[B20] Espina A (2003). Alexithymia in parents of daughters with eating disorders: its relationships with psychopathological and personality variables. J Psychosom Res.

[B21] Muller RJ (2000). When a Patient Has No Story To Tell: Alexithymia. Psychiatric Times.

[B22] Parker JD, Taylor GJ, Bagby RM (1989). The alexithymia construct: relationship with sociodemographic variables and intelligence. Compr Psychiatry.

[B23] Pasini A, Delle Chiaie R, Seripa S, Ciani N (1992). Alexithymia as related to sex, age, and educational level: results of the Toronto Alexithymia Scale in 417 normal subjects. Compr Psychiatry.

[B24] Costa PT, McCrae RR (1992). Professional manual revised NEO Personality Inventory (NEO-PI-R) and NEO Five-Factor (NEO-FFI) Inventory.

[B25] Fukunishi I, Nakagawa T, Nakamura H, Kikuchi M, Takubo M (1997). Is alexithymia a culture-bound construct? Validity and reliability of the Japanese versions of the 20-item Toronto Alexithymia Scale and modified Beth Israel Hospital Psychosomatic Questionnaire. Psychol Rep.

[B26] Digman JM (1990). Personality structure: emergence of the five-factor model. Annu Rev Psychol.

[B27] Kaiser HF (1960). The application of electronic computers to factor analysis. Educational and Psychological Measurement.

[B28] Cattell RB (1966). The scree test for the number of factors. Multivariate Behavioral Research.

[B29] Velicer WF (1976). Determining the number of components from the matrix of partial correlations. Psychometrika.

[B30] Browne MW, Cudeck R, Bollen KA Scott Long J (1993). Alternative ways of assessing model fit. Testing structural equation models.

[B31] Taylor GJ, Bagby RM, Ryan DP, Parker JD (1990). Validation of the alexithymia construct: a measurement-based approach. Can J Psychiatry.

[B32] Cole DA (1987). Utility of confirmatory factor analysis in test validation research. J Consult Clin Psychol.

[B33] Marsh HW, Balla JR, McDonald RP (1988). Goodness-of-fit indexes in confirmatory factor analysis: The effect of sample size. Psychol Bull.

[B34] Tuker LR, Lewis C (1973). The reliability coefficient for maximum likelihood factor analysis. Psychometrika.

[B35] Rybakowski J, Ziolkowski M, Zasadzka T, Brzezinski R (1988). High prevalence of alexithymia in male patients with alcohol dependence. Drug Alcohol Depend.

[B36] Akaike H (1974). A new look at the statistical model identification. IEEE Trans Autosomal Contr.

[B37] Schwarz G (1978). Estimating the dimension of a model. The Annals of Statistics.

[B38] Briggs SR, Cheek JM (1986). The role of factor analysis in the development and evaluation of personality scales. Journal of Personality.

[B39] Luminet O, Bagby RM, Wagner H, Taylor GJ, Parker JD (1999). Relation between alexithymia and the five-factor model of personality: a facet-level analysis. J Pers Assess.

[B40] Wise TN, Mann LS (1994). The relationship between somatosensory amplification, alexithymia, and neuroticism. J Psychosom Res.

[B41] Mann LS, Wise TN, Trinidad A, Kohanski R (1994). Alexithymia, affect recognition, and the five-factor model of personality in normal subjects. Psychol Rep.

[B42] Wise TN, Mann LS, Shay L (1992). Alexithymia and the five-factor model of personality. Compr Psychiatry.

[B43] Parker JDA, Bagby RM, Taylor GJ, Endler NS, Schmitz P (1993). Factorial validity of the 20-item Toronto Alexithymia Scale. Eur J Pers.

[B44] Erni T, Lotscher K, Modestin J (1997). Two-factor solution of the 20-item Toronto Alexithymia Scale confirmed. Psychopathology.

[B45] Loas G, Parker JD, Otmani O, Verrier A, Fremaux D (1997). Confirmatory factor analysis of the French translation of the 20-item Toronto Alexithymia Scale. Percept Mot Skills.

[B46] Luminet O, Bagby RM, Taylor GJ (2001). An evaluation of the absolute and relative stability of alexithymia in patients with major depression. Psychother Psychosom.

[B47] Honkalampi K, Koivumaa-Honkanen H, Tanskanen A, Hintikka J, Lehtonen J, Viinamaki H (2001). Why do alexithymic features appear to be stable? A 12-month follow-up study of a general population. Psychother Psychosom.

[B48] Lane RD, Sechrest L, Riedel R, Shapiro DE, Kaszniak AW (2000). Pervasive emotion recognition deficit common to alexithymia and the repressive coping style. Psychosom Med.

[B49] Kokkonen P, Veijola J, Karvonen JT, Laksy K, Jokelainen J, Jarvelin MR, Joukamaa M (2003). Ability to speak at the age of 1 year and alexithymia 30 years later. J Psychosom Res.

[B50] Erikson EH (1950). Childhood and Society.

[B51] Le HN, Berenbaum H, Raghavan C (2002). Culture and alexithymia: mean levels, correlates, and the role of parental socialization of emotions. Emotion.

